# The Leganés cognitive test correlates poorly with MRI evidence of global cortical atrophy in an underserved community: A population-based and nested case-control study in rural Ecuador (The Atahualpa Project)

**DOI:** 10.1590/S1980-57642014DN84000008

**Published:** 2014

**Authors:** Oscar H. Del Brutto, Robertino M Mera, Mauricio Zambrano, Julio Lama

**Affiliations:** 1 MD, School of Medicine, Universidad Espíritu Santo - Ecuador, Guayaquil, Ecuador. Department of Neurology, Hospital-Clínica Kennedy, Guayaquil, Ecuador; 2 MD, PhD, Gastroenterology Department, Vanderbilt University, Nashville, TN; 3 BS, Community Center, The Atahualpa Project, Atahualpa, Ecuador; 4 MD, Department of Imaging, Hospital-Clínica Kennedy, Guayaquil, Ecuador

**Keywords:** Leganés cognitive test, global cortical atrophy, population-based study, Atahualpa project, Ecuador

## Abstract

**Objective:**

We aimed to evaluate whether the Leganés cognitive test (LCT) correlates with global cortical atrophy (GCA) and can be used as a surrogate for structural brain damage.

**Methods:**

Atahualpa residents aged > 60 years identified during a door-to-door survey underwent MRI for grading GCA. Using multivariate generalized linear models, we evaluated whether continuous LCT scores correlated with GCA, after adjusting for demographics, education, cardiovascular health (CVH) status, depression and edentulism. In a nested case-control study, GCA severity was assessed in subjects with LCT scores below the cutoff level for dementia (< 22 points) and in matched controls without dementia.

**Results:**

Out of 311 eligible subjects, 241 (78%) were enrolled. Mean age was 69.2±7.5 years, 59% were women, 83% had primary school education, 73% had poor CVH status, 12% had symptoms of depression and 43% had edentulism. Average LCT score was 26.7±3, and 23 (9.5%) subjects scored < 22 points. GCA was mild in 108, moderate in 95, and severe in 26 individuals. On the multivariate model, mean LCT score was not associated with GCA severity (β=0.06, SE=0.34, p=0.853). Severe GCA was noted in 6 / 23 case-patients and in 8 / 23 controls (OR: 0.67, 95% CI: 0.14-2.81, p=0.752, McNemar's test).

**Conclusion:**

The LCT does not correlate with severity of GCA after adjusting for potential confounding variables, and should not be used as a reliable estimate of structural brain damage.

## Abstract (pt)

O teste cognitivo Leganés (TCL) é um instrumento para o rastreio rápido de demência em idosos com baixo nível educacional.

**Objetivo:**

Tivemos como objetivo avaliar se o TCL associa-se com medidas de atrofia cortical global (ACG) e pode ser usado como um substituto para a lesão cerebral estrutural.

**Métodos:**

Residentes de Atahualpa com idade > 60 anos identificados durante um levantamento porta-a-porta foram submetidos a ressonância magnética para avaliar a intensidade da ACG. Usando modelos lineares generalizados multivariados, avaliamos se escores TCL contínuos correlacionavam com a intensidade da ACG após ajustes para a dados demográficos, educação, saúde cardiovascular (CVH), depressão e edentulismo. Em um estudo caso-controle aninhado, avaliamos a gravidade da ACG em pessoas com escores no TCL abaixo do nível de corte para demência (< 22 pontos) e em pessoas pareados sem demência.

**Resultados:**

Dentre as 311 pessoas elegíveis, 241 (78%) foram selecionadas. A média de idade foi de 69,2±7,5 anos, 59% eram mulheres, 83% tinham o ensino primário, 73% tinham mau estado CVH, 12% apresentaram sintomas de depressão e 43% tinham edentulismo. Pontuações médias no TCL foram 26,7±3 e 23 (9,5%) pessoas tinham < 22 pontos. ACG foi leve em 108, moderada em 95 e grave em 26 pessoas. No modelo multivariado, a média de pontuação no TCL não foi associada com a gravidade da ACG (β=0,06, SE=0,34, p=0,853). ACG grave foi observada em 6 de 23 pacientes e em 8 de 23 controles (OR: 0,67; IC 95%: 0,14-2,81, p=0,752, teste de McNemar).

**Conclusão:**

O TCL não se associa com a gravidade da ACG após o ajuste para possíveis fatores de confusão e não deve ser usado como uma estimativa confiável de dano cerebral estrutural.

## INTRODUCTION

The burden of non-communicable diseases - including dementia - is steadily increasing in many low- and middle-income countries.^1^ People living in rural communities seem to be most vulnerable to these "new epidemics". In these regions, a process of epidemiologic transition is aggravated by lack of education, poor access to medical care and income issues that preclude people from affording the treatment of chronic diseases. Accurate estimates of the burden of dementia are essential for public health planning in these underserved populations. Such assessments, however, may be complicated by cross-cultural factors and illiteracy that render some of the most commonly used tests unreliable.^2^ In this context, it has been suggested that the Leganés Cognitive Test (LCT) is a reliable screening instrument for recognizing dementia in elderly persons living in low educated communities.^3-6^ There have been no attempts, however, to correlate cognitive performance on the LCT with neuroimaging studies to evaluate whether test scores reflect the severity of structural brain damage. We conducted a population-based, nested case-control study in community-dwelling elders living in Atahualpa - a village in rural Ecuador - to investigate whether LCT scores correlate with global cortical atrophy (GCA).

## METHODS

**Population studied**. Atahualpa is representative of the rural villages of coastal Ecuador. More than 95% of the population belongs to the Ecuadorian native/mestizo ethnic group (Amerindians). All inhabitants speak Spanish, and most have a low income. Most men work as carpenters, farmers or laborers, and almost all women are homemakers. The village has two elementary and one secondary school. The latter was opened less than 30 years ago, so most people aged > 40 years only have primary school education. A census performed by our group in June 2013 identified 2,478 Atahualpa residents, 311 (13%) of whom were aged > 60 years. Atahualpa is relatively isolated and closed. Inhabitants do not migrate, and a sizable proportion have never visited large urban centers (such as Guayaquil), which are more than 100 km away.

**Study design**. The Atahualpa Project is a population-based cohort study designed to reduce the increasing burden of non-communicable diseases in rural Ecuador.^7^ The protocol and the informed consent form were approved by the I.R.B. of Hospital-Clínica Kennedy, Guayaquil, Ecuador (FWA 00006867). For this part of the Atahualpa Project, trained rural doctors conducted a door-to-door survey to identify all Atahualpa residents aged > 60 years. We used different questionnaires designed to assess socio-demographic characteristics, cardiovascular health (CVH) status, symptoms of depression, severity of edentulism, and cognitive status of the population. In addition, participants were invited to undergo a brain MRI in Guayaquil.

**Covariates studied**. The CVH status of all participants was assessed by the use of the seven metrics proposed by the American Heart Association, including smoking status, body mass index, physical activity, diet, blood pressure, fasting glucose, and total cholesterol blood levels; each metric was categorized as ideal, intermediate, or poor, and the CVH status was classified as poor if at least one metric was in the poor range.^8^ Participants were also evaluated with the depression axis of the DASS 21, a consistent field instrument that quantitatively measures dysphoria, hopelessness, devaluation of life, self-deprecation, lack of interest/involvement, anhedonia and inertia, with seven questions rated on a four-point Likert scale ranging from 0 (not at all) to 3 (almost always) having a maximum total score of 21, defining a diagnosis of depression in individuals scoring > 5 points.^9^ For this evaluation, we used a Spanish version of the depression axis of the DASS-21 previously validated and used in Atahualpa's villagers.^10^ In addition, a rural dentist performed an oral exam with emphasis on the number of remaining teeth; individuals were classified into two groups according to whether they had severe edentulism - defined as those who had fewer than 10 remaining teeth - or otherwise.^11^

**Cognitive performance assessment**. The LCT was used for this evaluation, a reliable instrument (originally developed in Spanish) previously used and clinically validated in elderly persons living in poorly educated communities.^3-6^ The LCT mainly evaluates orientation and memory (Table 1), whose maximum score is 32 points where scores < 22 indicate dementia.

**Table 1:** Leganés Cognitive Test (original Spanish version).

**A. Sección de orientación (cada respuesta correcta es un punto. Máximo 8 puntos)**		
• ¿Qué fecha estamos?	Correcta [ ]	Incorrecta [ ]
• ¿Qué día de la semana es hoy?	Correcta [ ]	Incorrecta [ ]
• ¿Qué hora es?	Correcta [ ]	Incorrecta [ ]
• ¿En qué pueblo estamos?	Correcta [ ]	Incorrecta [ ]
• ¿Cuál es la dirección de su casa?	Correcta [ ]	Incorrecta [ ]
• ¿Cuántos años tiene?	Correcta [ ]	Incorrecta [ ]
• Fecha de nacimiento completa	Correcta [ ]	Incorrecta [ ]
• Nombre de la madre	Correcta [ ]	Incorrecta [ ]
**B. Sección de memoria (cada respuesta correcta es un punto. Máximo 24 puntos)**		
B1. Enseñarle a la persona 6 imágenes de objetos comunes (botella, camioneta, casa, árbol, sombrero, reloj)		
• B1a. Por cada respuesta correcta (viendo las imágenes), se otorga un punto (máximo 6)		Puntaje [ ]
• B1b. Luego de eso, pedirle a la persona que nombre los objetos que se le enseñó previamente (máximo 6 puntos)		Puntaje [ ]
• B1c. Luego de 5 minutos, pedirle a la persona que recuerde los objetos (máximo 6 puntos)		Puntaje [ ]
B2. Leerle a la persona una historia breve con 6 ideas		
(Calificar 1 punto x cada idea correcta, con máximo de 6 puntos)		Puntaje [ ]
Tres niños estaban solos en su casa / y la casa se quemó / Un bombero entró por la ventana / y se los llevó a un lugar seguro / Ninguno murió / pero algunos resultaron heridos.		

**Neuroimaging protocol**. Participants who had no contra-indications for undergoing MRI were transported to Guayaquil. All studies were performed with a Philips Intera 1.5T (Philips Medical Systems, the Netherlands) at Hospital-Clínica Kennedy. MRI included two-dimensional multi-slice turbo spin echo T1-weighted, fluid attenuated inversion recovery (FLAIR), T2-weighted, and gradient-echo sequences on the axial plane, as well as a T1-weighted sequence oriented on the sagittal plane. We used the pre-established brain imaging package delivered by the manufacturer to homogenize applicability by technicians; slice thickness was 5 mm with 1 mm gap between slices.

**Figure 1:**
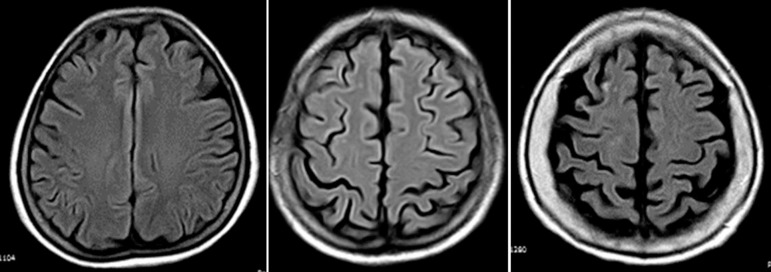
Fluid attenuated inversion recovery MRIs (TR 9000, TE 120, TI 2500) showing severity of global cortical atrophy (GCA) according to Pasquier et al. From left to right, columns represent mild, moderate and severe global GCA, respectively. In mild GCA there is sulcal opening peripherally, moderate GCA is characterized by widening along the length of the sulci, and severe GCA is present when there is gyral thinning.

A neurologist (OHD) and a neuroradiologist (JL) independently read all MRIs, blinded to clinical data. Primary interest was focused on the presence of GCA, using the visual scale proposed by Pasquier et al. (1996) (Figure 1). Kappa coefficients for inter-rater agreement (n=258) were 0.76 for severity of GCA, and disagreements were resolved by consensus.

**Statistical analysis**. All analyses were carried out using STATA version 13 (College Station, TX, USA) software. Descriptive statistics were expressed as means±standard deviations for continuous variables and as percentages with 95% CI for categorical variables. A p-value of less than 0.05 was considered significant. Using a multivariate generalized linear model, the association between GCA and LCT score was examined (in all participants) after adjusting for demographics, education, CVH status, edentulism and depression. Subsequently, the presence of severe GCA was compared in subjects with an LCT score below the recommended cutoff for dementia (< 22 points) and in a similar number of controls (LCT > 22) matched for age, sex, education, CVH status, depression and edentulism, using McNemar's test for correlated proportions (matched-pair analysis).

## RESULTS

The door-to-door survey identified 311 Atahualpa residents aged > 60 years. Of these, 258 (83%) underwent a brain MRI. Reasons for not obtaining MRI included refusal to participate (n=26), severe disability (n=11), claustrophobia (n=8), and implanted pacemaker (n=1); a further seven individuals had either died or emigrated between the time of the survey and invitation. Seventeen of the 258 subjects who underwent MRI were unable to perform the LCT due to aphasia or severe visual or hearing impairment. Therefore, the present study included 241 individuals. Mean age of subjects was 69.2±7.5 years, 141 (59%) were women and 199 (83%) had primary school education only. A poor CVH status was noted in 175 (73%) participants and the mean±SD number of poor metrics per person was 1.2±1. Thirty individuals (12%) had symptoms of depression and 104 (43%) had severe edentulism. Mean score on the LCT was 26.7±3 points, and 23 participants scored < 22 points. GCA was mild in 108, moderate in 95, and severe in 26 subjects. As only 12 participants had no GCA, they were grouped with subjects exhibiting mild GCA for analysis. Univariate analysis showed that individuals with moderate-to-severe GCA were older, less educated and more often edentulous than those with none-mild GCA (Table 2). On the multivariate generalized linear model, mean LCT score was not associated with GCA severity (β=0.06, SE=0.34, p=0.853). In the nested case-control study, severe GCA was noted in 6/23 case-patients and in 8/23 controls (OR: 0.67, 95% CI: 0.14-2.81, p=0.752).

**Table 2:** Characteristics of community-dwelling elders living in Atahualpa according to severity of global cortical atrophy (GCA).

	Total series n=241	None-mild GCA n=120	Moderate GCA n=95	Severe GCA n=26	Significance
Age, mean±SD years	69.2±7.5	64.8±3.5	72.1±7.3	79.2±7.5	0.0001
Women, n (%)	141 (59%)	70 (58%)	53 (56%)	18 (69%)	0.468
Up to primary School, n (%)	199 (83%)	90 (75%)	86 (91%)	23 (88%)	0.008
Poor CVH status, n (%)	175 (73%)	84 (70%)	68 (72%)	23 (88%)	0.153
Symptoms of depression, n (%)	30 (12%)	14 (12%)	13 (14%)	3 (12%)	0.726
Severe edentulism, n (%)	104 (43%)	41 (34%)	49 (52%)	14 (54%)	0.019

## DISCUSSION

Results of the present study showed that LCT scores were not associated with severity of GCA after adjusting for a number of confounding variables, and suggest that this field instrument should not be used as an estimate of structural brain damage in low educated elders. This lack of association was also noted in the nested study upon comparing the occurrence of severe GCA in individuals with LCT scores below the recommended cutoff for dementia and in matched controls.

The LCT was originally introduced as an aid for cognitive screening of elders with low levels of education living in a small satellite city near Madrid, Spain.^3^ In the original publication as well as in subsequent studies, the LCT had been tested against other screening instruments such as Folstein's Mini-Mental State Examination, but no attempt to correlate LCT results with neuroimaging findings has been made.

The present study has several limitations. We relied on a visual rating scale and did not use volumetric assessment of cortical grey matter. In addition, medial temporal atrophy was not evaluated, one of the earliest markers of Alzheimer's disease and other dementias.^12^ However, the population-based and nested case-control design, together with the homogeneous characteristics of Atahualpa's residents and the models used for assessing the association between GCA and LCT scores, contribute to the merit of the study results. Further studies are needed to determine whether scores on the LCT correlate with MRI findings suggestive of structural brain damage.
